# Emergent Alcohol Septal Ablation for Left Ventricular Tract Obstruction in 2 Patients

**DOI:** 10.1016/j.cjco.2024.06.008

**Published:** 2024-06-25

**Authors:** Agnese Vella, Georgios Giannakopoulos, Nils Perrin, Martin Nicoletti, Stephane Noble

**Affiliations:** a Structural Heart Unit, Service of Cardiology, Department of Medicine, Geneva University Hospital and University of Geneva, Geneva, Switzerland; b Department of Radiology, Lausanne University Hospital and University of Lausanne, Lausanne, Switzerland

**Left ventricular outflow tract obstruction (LVOTO), defined by a peak instantaneous gradient at the level of the left ventricular outflow tract of at least 30 mm Hg, and becoming hemodynamically significant when the gradient ≥ 50 mmHg, is a common cause of unexplained refractory hypotension. A dynamic phenomenon, typically associated with hypertrophic cardiomyopathy, it can also result from a combination of various anatomic and physiological factors. Two cases following non-ST-elevation myocardial infarction and transcatheter aortic valve replacement (TAVR), unresponsive to standard LVOTO treatment, improved after emergent alcohol septal ablation (ASA).**

## Case Presentations

### Case 1

**Figure 1:**
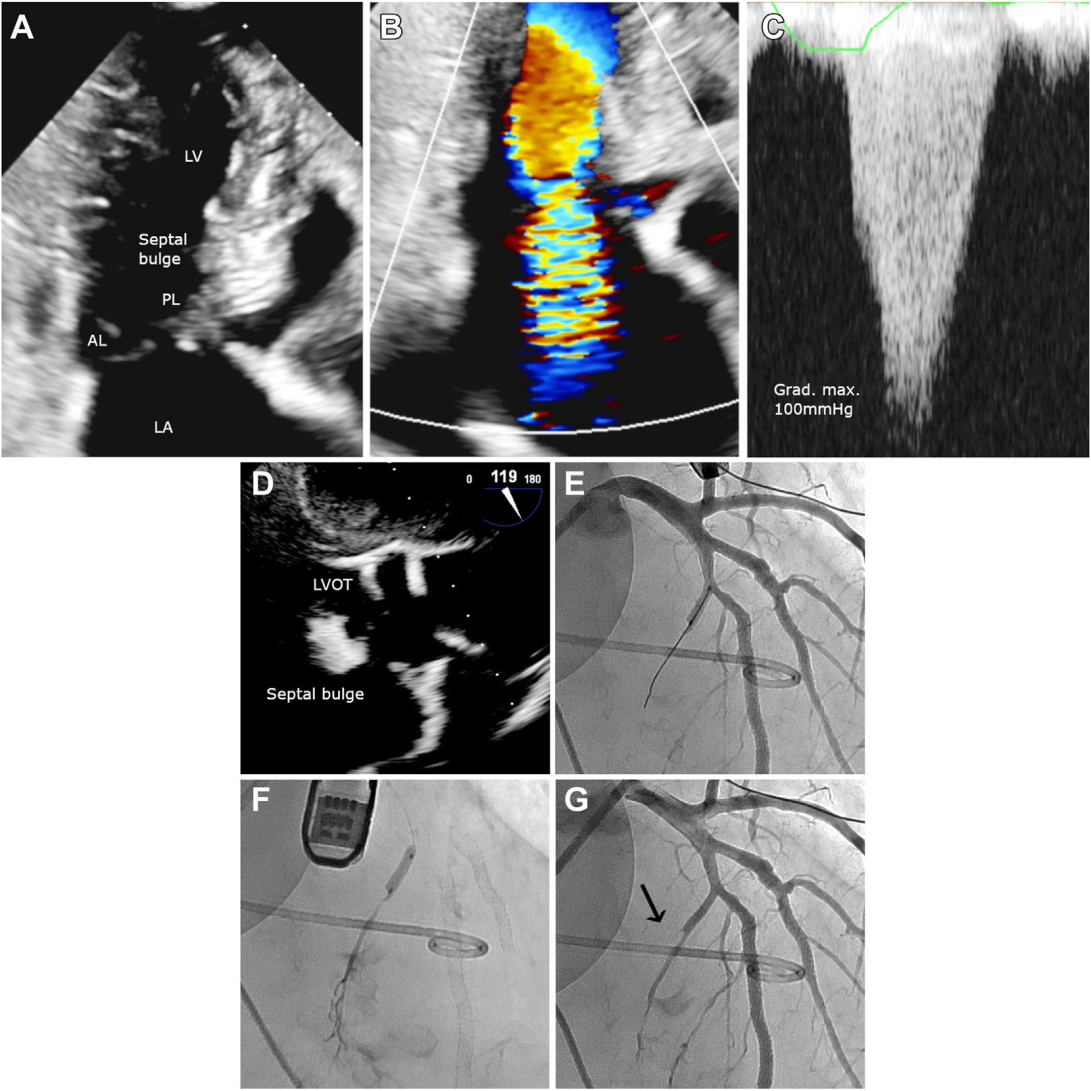
Transthoracic echocardiography after coronary revascularization and initiation of supportive treatment (**top**) and alcohol septal ablation under transesophageal echocardiographic guidance (**bottom**). (**A**) Systolic anterior motion phenomenon. (**B**) Severe mitral regurgitation. (**C**) "Dagger-shaped" envelope indicative of left ventricular outflow tract (LVOT) obstruction. (**D**) Transesophageal echocardiography with aortic valve long-axis view (119° probe) and contrast enhancement to localize the myocardium perfused by the septal artery. (**E**) Catheterization of the first septal perforator artery. (**F**) Ethanol injection in the first septal perforator artery. (**G**) Final result with no flow in the first septal perforator artery (**arrow**). AL, mitral anterior leaflet; Grad, gradient; LA, left atrium; LV, left ventricle; max, maximum; PL, mitral posterior leaflet.

A 78-year-old woman with no significant past medical history was brought to the emergency department for dizziness with persistent hypotension. Clinical examination revealed that she had a blood pressure of 56/49 mm Hg, heart rate of 119 beats per minute, an oxygen saturation of 78%, and signs of peripheral hypoperfusion, with a systolic murmur graded 3/6. Laboratory data showed the following: an elevated high-sensitivity cardiac troponin T level, with significant change on repeat measurements (63-187-2943 ng/L; normal, < 14 ng/L); a creatine kinase-MB level of 1190 U/L; normal, 47-222 U/L), and an N-terminal pro-brain natriuretic peptide level of 2281 ng/L (normal, < 300 ng/L). Her kidney function and electrolyte levels were within normal ranges. A 12-lead electrocardiogram showed new atrial fibrillation at 119 beats per minute, with anterolateral ST depression. Transthoracic echocardiography (TTE) revealed the following: normal left ventricular (LV) size with moderate concentric hypertrophy (LV end-diastolic diameter: 4 cm; end-diastolic interventricular septal thickness: 1.4 cm; LV posterior wall thickness: 1.3 cm); normal LV ejection fraction (65%-70%), with anterior and anterolateral wall-motion hypokinesis. Dynamic LVOTO, systolic anterior motion phenomenon, and moderate-to-severe mitral regurgitation were observed. Intravenous hydration, metoprolol, and continuous positive airway pressure were initiated. Coronary angiography revealed a 70% stenosis in the left anterior descending artery, and a 90% stenosis of the first diagonal artery, both of which were treated with stent implantation, given the chest pain with elevated cardiac biomarkers, electrocardiogram modification, and anterior hypokinesis. Despite revascularization and supportive treatment (intravenous hydration and metoprolol), the patient presented refractory acute pulmonary edema. Repeat TTE showed an LVOTO with > 100 mm Hg of maximal gradient, with the classic “dagger-shaped” envelope, and worsening severity of mitral regurgitation (Fig. 1; Videos 1-3 , view videos online). An emergent ASA was performed in the next 24 hours under transesophageal echocardiographic guidance (Video 4 , view video online), and 1.5 ml of 98% dehydrated ethanol was injected into the first septal perforator artery (Fig. 1). Immediate hemodynamic invasive assessment revealed a negative Brockenbrough-Braunwald-Morrow sign. TTE at day 6 showed a significant LVOTO reduction (aortic maximal gradient at rest: 16 mm Hg; aortic maximal gradient during the strain phase of the Valsalva maneuver: 24 mm Hg), with mild-to-moderate mitral regurgitation, and no more systolic anterior motion phenomenon. The 6-month TTE assessment was similar, and the patient was asymptomatic.

### Case 2

**Figure 2:**
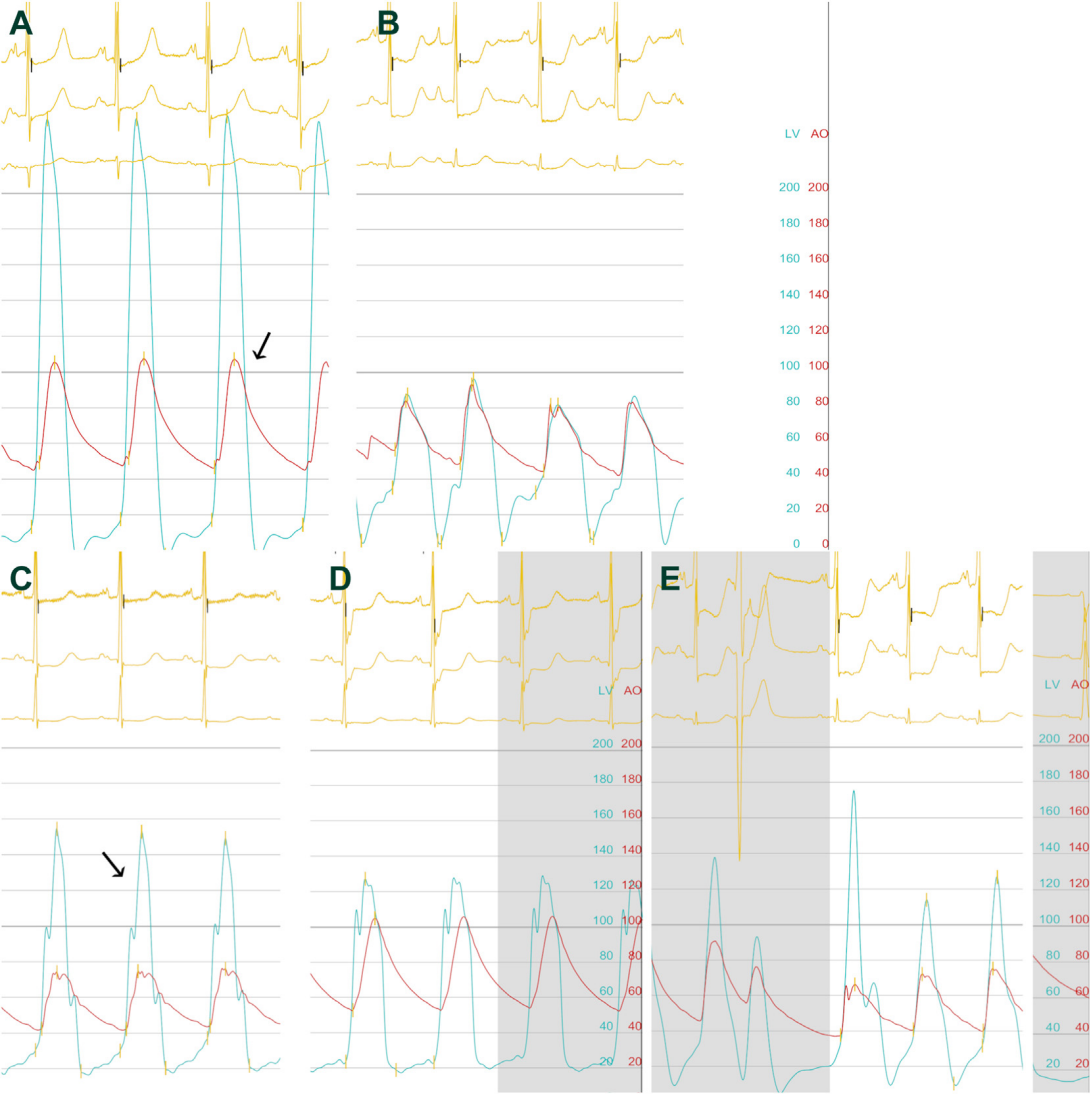
Left ventricle–aorta hemodynamic assessment. (**A**) Before transcatheter aortic valve replacement: mean gradient of 68 mm Hg, with pulsus tardus and parvus pattern of the aortic pressure curve (**arrow**). (**B**) After transcatheter aortic valve replacement, immediate result with no significant mean gradient. (**C**) Before alcohol septal ablation: left ventricular outflow tract obstruction with “dagger-shaped” envelope (**arrow**), “spike and dome” configuration (**red**), and increased peak-to-peak gradient of 76 mm Hg. (**D**) After alcohol septal ablation: reduction of left ventricular outflow tract obstruction and peak-to-peak gradient to 15 mm Hg. (**E**) Positive Brockenbrough-Braunwald-Morrow sign, defined by a rise of left ventricular pressure (**blue**), due to an increase of ventricular contractility after premature ventricular contraction, with a drop in the arterial pulse pressure (**red**).

An 86-year-old woman with a history of hypertension and ischemic stroke was referred for TAVR for symptomatic severe aortic stenosis (AS; aortic valve area: 1 cm^2^; mean gradient, 50 mm Hg; peak aortic velocity, 5 m/s at TTE). Preprocedural TTE revealed a small LV cavity size, with asymmetrical severely thickened walls (LV end-diastolic diameter: 3.3 cm; end-diastolic interventricular septal thickness: 1.6 cm; LV posterior wall thickness: 1.3 cm), and normal LV ejection fraction (65%), with significant dynamic LVOTO (maximal gradient: 60 mm Hg at rest). Invasive hemodynamic assessment confirmed the AS severity, with a characteristic tardus-parvus pattern of the aortic curve (Fig. 2A). The patient underwent a 26-mm self-expandable (Evolut PRO +, Medtronic, Minneapolis, MN) valve implantation with a 3-mm Hg final mean gradient, a peak-to-peak gradient of 22 mm Hg, and no paravalvular regurgitation. The postimplantation simultaneous intraventricular and supravalvular invasive hemodynamic assessment revealed a dynamic LVOTO with a positive Brockenbrough-Braunwald-Morrow sign (Fig. 2, B and E). Vasopressor injection by the anesthesiologists, due to slight hypotension, led to hemodynamic collapse with severe arterial hypotension. TTE showed subaortic acceleration starting at the basal septum, with LVOTO reaching 100 mm Hg at rest, systolic anterior motion phenomenon, and moderate mitral regurgitation. The invasive hemodynamic assessment showed an increased peak-to-peak gradient of 76 mm Hg, and a “spike and dome” configuration of the aortic pressure waveform, consistent with LVOTO (Fig. 2C). An ASA was performed on day 2, because of persistent severe hypotension despite intravenous beta-blocker and volume replenishment. An injection of 2 ml of 98% dehydrated ethanol was made into the first septal perforator artery, with an immediate hemodynamic improvement and significant reduction in peak-to-peak gradient (15 mm Hg; Fig. 2D). The 6-month TTE showed a nonsignificant LV tract gradient (19 mm Hg at rest), with a minimal increase during a Valsalva maneuver (26 mm Hg), and an aortic valve mean gradient of 11 mm Hg.

## Discussion

We report 2 cases of dynamic LVOTO that were treated successfully by emergent ASA. LVOTO is defined by a peak instantaneous gradient at the level of the LVOT of ≥ 30 mm Hg (at rest or with provocation), and it is considered hemodynamically significant when the gradient ≥ 50 mm Hg. LVOTO might be accentuated by systolic anterior motion of the anterior mitral leaflet protruding into the left ventricular outflow tract during systole. LVOTO should be suspected in cases of unexplained hypotension that is refractory to inotropic. TTE is the primary diagnostic tool. In situations in which diagnostic uncertainty persists, additional modalities, such as invasive pressure measurement, transesophageal echocardiography, and magnetic resonance imaging, may provide valuable insights.

LVOTO is a dynamic phenomenon that requires the coexistence of anatomic factors and physiological conditions, and it typically is associated with hypertrophic cardiomyopathy.^1^ However, LVOTO also might either occur in the context of acute myocardial infarction or develop in patients with acute preload or afterload reduction. In the setting of acute myocardial infarction, the compensatory hyperkinesia of the normally perfused myocardial segments promotes LVOTO, especially with preexisting septal hypertrophy, as demonstrated by our first case.

In cases of AS, as shown by our second case, the sudden afterload reduction induced by TAVR may lead to LVOTO, inducing the so-called “suicidal left ventricle,“ defined as severe hypotension causing hemodynamic collapse, with or without end-organ failure,^2^ a rare, but life-threatening complication associated with increased in-hospital mortality and complications,^3^ Female sex, small ventricle, prominent septal bulge, and hyperdynamic contractility are risk factors for LVOTO, which potentially can be underestimated in preprocedural assessment because of the increased afterload on the LV.^2^

Our cases are consistent with other reports in the literature of emergent ASA being performed successfully to manage acute LVOTO and improve hemodynamic stability. Initial management is focused on fluid resuscitation, use of negative chronotropic agents, and avoidance of inotropic agents. A temporary right ventricular pacemaker can help obtain desynchrony to reduce LVOTO. If the condition is unresponsive, invasive management (ASA or myomectomy) has been associated with better outcomes^4^; indeed, mitral transcatheter edge-to-edge repair also could be an option.^5^ ASA is the preferred approach for relieving dynamic LVOTO in patients who have a high level of surgical risk, with concomitant myocardial hypertrophy and AS.^1^^,^^6^ Which patients are at risk of developing LVOTO after TAVR, and who may benefit from prophylactic ASA before the procedure, remain unclear.^6^^,^^7^ The possibility of revealing dynamic LVOTO during TAVR should be weighed carefully in patient selection for the procedure.^7^

In cases of failure of conventional LVOTO treatment, post-TAVR or post–non-ST segment elevation myocardial infarction, invasive management using ASA can improve severe refractory LVOTO and clinical outcomes.

**Novel Teaching Points**

• LVOTO should be considered a cause for unexplained refractory hypotension.
• Acute myocardial infarction may promote LVOTO in the context of preexisting septal hypertrophy.
• Aortic valvular replacement for AS, associated with a prominent septal bulge, may unmask dynamic LVOTO, and carries a risk of development of hemodynamic instability after TAVR (“suicidal ventricle”).
• Preoperative TTE may help in anticipating the "suicidal ventricle" phenomenon after TAVR.
• In selected patients, emergent ASA can improve LVOTO and patient outcomes.

## Supplementary Material

Video 1 Transthoracic echocardiography with a three-chamber view demonstrating systolic anterior motion phenomenon.

Video 2 Transthoracic echocardiography with a three-chamber view and zoom on mitral valve demonstrating systolic anterior motion phenomenon.

Video 3 Transthoracic echocardiography with a three-chamber view and color Doppler revealing severe mitral regurgitation.

Video 4 Transesophageal echocardiography with aortic valve long-axis view (106° probe) and contrast enhancement to localize the myocardium perfused by the septal artery.
